# Mapping QTLs Controlling Flowering Time and Important Agronomic Traits in Pearl Millet

**DOI:** 10.3389/fpls.2017.01731

**Published:** 2017-12-20

**Authors:** Sushil Kumar, C. Tom Hash, T. Nepolean, C. Tara Satyavathi, Govind Singh, Mahesh D. Mahendrakar, Rattan S. Yadav, Rakesh K. Srivastava

**Affiliations:** ^1^Plant Biotechnology Centre, Swami Keshwanand Rajasthan Agricultural University, Bikaner, India; ^2^International Crops Research Institute for the Semi-Arid Tropics, Patancheru, India; ^3^Centre of Excellence in Biotechnology, Anand Agricultural University, Anand, India; ^4^International Crops Research Institute for the Semi-Arid Tropics, Niamey, Niger; ^5^Indian Agricultural Research Institute, New Delhi, India; ^6^Institute of Biological, Environmental and Rural Sciences, Aberystwyth University, Aberystwyth, United Kingdom

**Keywords:** RILs, QTL, flowering time, grain weight, plant height, pearl millet

## Abstract

Pearl millet [*Pennisetum glaucum* (L.) R. Br.] is a staple crop for the people of arid and semi-arid regions of the world. It is fast gaining importance as a climate resilient nutricereal. Exploiting the bold seeded, semi-dwarf, and early flowering genotypes in pearl millet is a key breeding strategy to enhance yield, adaptability, and for adequate food in resource-poor zones. Genetic variation for agronomic traits of pearl millet inbreds can be used to dissect complex traits through quantitative trait locus (QTL) mapping. This study was undertaken to map a set of agronomically important traits like flowering time (FT), plant height (PH), panicle length (PL), and grain weight (self and open-pollinated seeds) in the recombinant inbred line (RIL) population of ICMB 841-P3 × 863B-P2 cross. Excluding grain weight (open pollinated), heritabilities for FT, PH, PL, grain weight (selfed) were in high to medium range. A total of six QTLs for FT were detected on five chromosomes, 13 QTLs for PH on six chromosomes, 11 QTLs for PL on five chromosomes, and 14 QTLs for 1,000-grain weight (TGW) spanning five chromosomes. One major QTL on LG3 was common for FT and PH. Three major QTLs for PL, one each on LG1, LG2, and LG6B were detected. The large effect QTL for TGW (self) on LG6B had a phenotypic variance (*R*^2^) of 62.1%. The *R*^2^ for FT, TGW (self), and PL ranged from 22.3 to 59.4%. A total of 21 digenic interactions were discovered for FT (*R*^2^ = 18–40%) and PL (*R*^2^ = 13–19%). The epistatic effects did not reveal any significant QTL × QTL × environment (QQE) interactions. The mapped QTLs for flowering time and other agronomic traits in present experiment can be used for marker-assisted selection (MAS) and genomic selection (GS) breeding programs.

## Introduction

Pearl millet [*Pennisetum glaucum* (L.) R. Br.] is a staple crop for the arid and semi-arid tropics of Asia and Africa (Anuradha et al., 2017). It is an inexpensive source of nutrients like carbohydrates, proteins, vitamins, and minerals compared to many cereals (rice, wheat, maize, etc.) and pulses (chickpea, pigeonpea, green gram, black gram, etc.) in these regions. Pearl millet is also valued for its quality fodder, high biomass, and greater per day productivity. Globally, pearl millet is cultivated on more than 29 million hectares of the arid and semi-arid regions of Africa (16 Mha), Asia (11 Mha), and Latin America (http://www.cgiar.org/our-strategy/crop-factsheets/millets/).

In the Indian subcontinent and sub-Saharan Africa, water stress occurs frequently owing to scanty and erratic rains during the cropping season leading to a post-flowering moisture stress (Yadav et al., 2011). Though substantial improvement has been achieved for both grain and fodder yield, and seed yield stability in pearl millet (Yadav et al., 2003), the cultivable area under pearl millet is reducing. The pace of genetic improvement of quantitative traits is slow due to integration of many physiological processes, complex inheritance and genotype × environment interactions (GEI). This suggested that intensification of breeding programs is needed to increase grain yield potential of pearl millet to cap the growing demands of millets in rural as well as in urban areas.

Poor sink capacity and low harvest index are the inherent bottlenecks of pearl millet (Yagya and Bainiwal, 2001) mainly due to lower seed weight. Therefore, it is important to increase seed size to boost the seed productivity and grain yield. Understanding the genetics of agronomic traits like flowering time, plant height, and yield-related traits *viz*. 1,000-grain weight, and panicle length are required for a successful breeding program. Molecular genetics and genomic tools have been used in pearl millet to identify QTLs for various traits ranging from agronomic importance to stress resistance (Yadav et al., 2011) and for domestication (Poncet et al., 2000, 2002). However, QTL analysis for yield determining traits like flowering time, plant height, panicle length, and 1,000-grain weight has been sparingly studied. This study was undertaken with an objective of extending the present understanding of the underlying QTLs for the important adaptation and agronomic traits, leading to enhanced efficiency and precision of the pearl millet breeding.

## Materials and methods

A set of 120 entries (106 F_6_ recombinant inbred line (RIL) population + 2 parents + 4 checks) were raised in experimental plots in a three replication alpha-lattice design for two seasons. The first environment (E1) consisted of late *Kharif (an Indian term for rainy season*) 2009; while the second environment E2 was summer 2010. The RILs were derived from a cross between ICMB 841-P3 and 863B-P2 as described in Kumar et al. (2016). The downy mildew resistant female genotype ICMB 841 was developed by pure-line selection, while the male line 863B was produced from an *Iniadi* landrace germplasm from the Togolese Republic. The standard agronomic management was performed to grow a vigorous crop. The RILs were phenotyped for flowering time (FT), plant height (PH), panicle length (PL), and 1,000-grain weight (TGW) in each plot in each field experiment. FT was scored as the number of days from the date of sowing until 50% of the plants in each plot exerted stigmas on their main stem panicles. PH was recorded at maturity on three competitive plants per plot as the average value of the distance (cm) from the soil surface to the top of the panicle on the main stem. PL was measured (in cm) as the length from base to the tip of panicle on the main stem of the same three plants considered for plant height in each plot, and the average of these three values was recorded. The weight of 1,000 randomly-selected grains (TGW, gm), for self (TGW_self) from selfed earheads and open pollinated (TGW_OP) from open pollinated earheads were determined in three replications per plot and the mean of these three observations was multiplied by ten to estimate the weight of 1,000 grains. Residual maximum likelihood (ReML) algorithm with a mixed model was used to find the best linear unbiased predictions (BLUPs) in GenStat for Windows (12th Edition) (Payne et al., 2009). Plot-means basis broad-sense heritability (H^2^) was determined as per Falconer (1989) using PROC MIXED in SAS (SAS Institute Inc, 1999). Phenotypic and genotypic correlations were estimated using GenStat for Windows (12th Edition).

A recently reported RIL population-based linkage map of the same cross developed at ICRISAT, India as described in Kumar et al. (2016) was used for QTL mapping. In brief, this map comprises of 95 microsatellite markers (SSRs), 2 STS markers and 208 DArT markers. The information of development of SSRs and DArT markers are reported in Qi et al. (2001), Allouis et al. (2001), Budak et al. (2003), Qi et al. (2004), Senthilvel et al. (2008), Rajaram et al. (2013), and Supriya et al. (2011). The map had an average inter-marker distance of 5.7 cM, with a total map length of 1,748.7 cM spanning 7 linkage groups. The LG6 was broken into three linkage groups. Genetic distances were calculated in MAPMAKER/EXP 3.0 program (Lander et al., 1987) using Haldane mapping function.

Correlation analysis was performed with PROC CORR in SAS to obtain phenotypic association among traits. QTL analysis was performed by PLABQTL software (Utz and Melchinger, 1996) through composite interval mapping (CIM) method with 2 cM walk speed to detect putative QTLs on the linkage groups. A minimum log of the odds (LOD) threshold of 3.0 was employed to declare the presence of significant QTL by accounting for the Bonferroni correction. QTL × QTL × environment (Q × Q × E) interaction was computed in QTLNetwork 2.1 software (Yang et al., 2008).

## Results

### Performance of the population

The mean performance and the descriptive statistics of studied traits in the RIL population with both parents raised in 2009 and 2010 are presented in Table 1. Except for PL, ICMB 841-P3 exhibited significantly lower BLUPs than 863B-P2 for all observed agronomic traits across the two environments. The difference between parental BLUPs for FT and PL was recorded as significant in both of the individual screening environments. Similarly, the BLUPs of TGW of self (TGW_self) and open-pollinated (TGW_OP) seeds between the parents was non-significant in E1 and E2, respectively. However, in the pooled analysis the parental differences for both traits were significant.

**Table 1:** Descriptive statistics of phenotypic values observed in the (ICMB 841-P3 × 863B-P2)-derived RIL population and their parental lines in two different environments (E1 = late *Kharif* 2009; E2 = Summer 2010) at ICRISAT-Patancheru, and across these two environments.

**Trait**	**Environment**	**ICMB 841 (P1)**	**863B (P2)**	**RILs**	**RILs**	**P1 vs. P2**	**P1 vs. RILs**	**P2 vs. RILs**
		**BLUP**	**BLUP**	**BLUP**	**RANGE**	**Pr > F**	**Pr > F**	**Pr > F**
FT	2009	43.01 ± 0.54	46.85 ± 0.53	46.02 ± 0.09	37.21−53.43	^**^	^**^	ns
	2010	43.88 ± 0.46	46.28 ± 0.46	45.90 ± 0.08	36.47−54.81	^**^	^**^	ns
	Across	43.72 ± 1.14	46.43 ± 1.14	45.96 ± 0.18	36.2−54.18	^**^	^*^	ns
PH	2009	94.41 ± 2.43	108.40 ± 2.39	110.76 ± 0.41	84.26−128.89	^**^	^**^	ns
	2010	100.81 ± 2.70	108.71 ± 2.69	119.02 ± 0.45	90.83−138.76	^*^	^**^	^**^
	Across	99.28 ± 5.47	109.64 ± 5.47	114.83 ± 4.12	76.06−131.60	^*^	^**^	ns
PL	2009	17.38 ± 0.49	18.32 ± 0.48	18.50 ± 0.08	13.85−23.30	ns	^*^s	ns
	2010	17.86 ± 0.55	19.20 ± 0.54	20.28 ± 0.09	14.45−26.57	ns	^**^	ns
	Across	17.90 ± 1.11	18.73 ± 1.11	19.38 ± 0.84	13.8−25.13	ns	^*^	ns
TGW_self	2009	7.01 ± 0.35	9.28 ± 0.35	8.54 ± 0.06	6.2−11.96	^**^	^**^	^*^
	2010	7.08 ± 0.24	7.72 ± 0.24	7.15 ± 0.04	4.76−10.48	ns	ns	^*^
	Across	7.02 ± 0.81	8.58 ± 0.81	7.84 ± 0.68	5.54−10.86	^*^	ns	ns
TGW_OP	2009	7.20 ± 0.30	7.55 ± 0.29	7.66 ± 0.05	6.49−9.40	ns	ns	ns
	2010	8.14 ± 0.28	10.74 ± 0.28	8.17 ± 0.05	6.26−11.83	^**^	ns	^**^
	Across	7.65 ± 0.46	8.93 ± 0.46	7.92 ± 0.28	3.01−10.63	^*^	ns	^**^

FT, Time to 50% flowering (d); PH, Plant height (cm); PL, Panicle length (cm); TGW_self, Self-pollinated 1,000-grain weight (g); TGW_OP, Open-pollinated 1,000-grain weight (g);

* Significant at 5% level;

** *Significant at 1% level; ns, non-significant*.

A wide range of variation among RILs was also displayed for traits studied in both (E1 and E2) the environments. Higher estimates of BLUPs of RILs for PH, PL, and TGW_OP were observed in E2, though BLUPs for FT and TGW_self were higher in E1. The BLUPs of ICMB 841-P3 were significantly lower for FT, PH, TGW_self, and TGW_OP traits compared to 863B-P2. Pooled environment analyses revealed that mean BLUPs of TGW_selfed and TGW_OP of ICMB 841-P3 and RILs were non-significantly different. The average performances of the RIL population for FT and PL were non-significantly dissimilar from 863B-P2 over the environments; while PH and TGW_OP seeds of RILs in E1 were similar to 863B-P2. The differences in BLUPs of RILs and male parent were significant for TGW_OP seeds in pooled data analysis.

### Variance components

Higher genotypic variances were recorded for FT and TGM_self in E1 compared to E2. However, remaining traits demonstrated higher genotypic variance in E2 (Table 2). Variances due to genotypes for traits were significant (at *P* < 0.01) in across environment analysis. Similarly, variances due to genotype × environment interaction (GEI) were significant (at *P* < 0.01) for all traits across two environments. Nevertheless, as compared to GEI variance, genetic variances were significantly higher (and often an order of magnitude larger) for all the studied traits.

**Table 2:** Genotypic variances (σ^2^g), G×E interaction variances (σ^2^g×E), standard errors (SE) and operational heritabilities (H^2^, broad-sense) for traits observed in the (ICMB 841-P3 × 863B-P2)-derived RIL population, in two different environments at ICRISAT-Patancheru (E1 = late *Kharif* 2009; E2 = summer 2010), and across these two environments.

**Trait**	**E1**			**E2**			**Pooled**				
	**σ^2^g**	**SE**	***H*^2^**	**σ^2^g**	**SE**	***H*^2^**	**σ^2^g**	**SE**	**σ^2^g × E**	**SE**	***H*^2^**
FT	13.84	2.05	0.86	11.11	1.63	0.87	9.85	1.63	2.73	0.48	0.78
PH	82.68	13.98	0.63	137.40	22.15	0.70	84.84	15.08	24.6	6.21	0.55
PL	4.07	0.67	0.68	6.58	1.04	0.73	4.42	0.74	0.92	0.24	0.65
TGW_self	1.69	0.29	0.62	1.26	0.20	0.72	1.12	0.20	0.38	0.09	0.58
TGW_OP	0.68	0.14	0.47	1.17	0.20	0.65	0.63	0.13	0.27	0.07	0.46

*FT, Time to 50% flowering (d); PH, Plant height (cm); PL, Panicle length (cm); TGW_self, Self-pollinated 1,000-grain weight (g); TGW_OP, Open-pollinated 1,000-grain weight (g*).

### Heritability, correlation analysis, and frequency distributions

FT was highly heritable in both E1 and E2, while PL and TGW_self were highly heritable in E2 (*H*^2^ = >0.70) on Robinson's scale (Robinson et al., 1949). In the pooled analysis, PH, PL, and TGW_self had medium heritabilities. A decrement in H^2^ was detected after dividing variance due to GEI for the combined dataset across 2009 and 2010 (Table 2). In the joint analysis, H^2^ ranged between 0.46 (TGW_OP) and 0.78 (FT). FT was the most heritable observed trait in both the environments.

Genetic and phenotypic correlations were analyzed among five traits. The genotypic and phenotypic correlation analysis showed that days to flowering was positively and significantly associated with PH (gc = 0.317, pc = 0.184). However, FT had a significant negative relationship (gc = −0.257, pc = −0.154) with TGW. PH showed positively significant association with PL in across these two screening environments (gc = 0.589, pc = 0.488). The phenotypic correlation coefficients were significantly negative between plant height and TGW (−0.137) while genotypic correlation was non-significantly negative. The correlations were significantly positive between TGW and TGW_OP (gc = 0.858). A moderate and positive significant phenotypic correlation (pc = 0.347) was detected between TGW_OP and TGW_self. The coefficients of genotypic correlation were higher compared to phenotypic level correlation coefficients for studied characteristics (Table 3). In comparison to phenotypic, higher genotypic correlations suggested that genotype are having high interaction with the environment.

**Table 3:** Genotypic and phenotypic correlations^A^ between trait BLUPs across two environments (late *Kharif* 2009 and summer 2010) at ICRISAT-Patancheru in the pearl millet RIL population based on the cross (ICMB 841-P3 × 863B-P2).

**Trait**	**FT**	**PH**	**PL**	**TGW_self**	**TGW_OP**
FT	1	0.184^**^	−0.112^**^	−0.154^**^	−0.072
PH	0.317^**^	1	0.488^**^	−0.137^**^	−0.018
PL	−0.125	0.589^**^	1	−0.118^**^	−0.008
TGW_self	−0.257^**^	−0.115	−0.017	1	0.347^**^
TGW_OP	−0.1	−0.136	−0.097	0.858^**^	1

A Genotypic correlation below the diagonal; phenotypic correlations above the diagonal. FT, Time to 50% flowering (d); PH, Plant height (cm); PL, Panicle length (cm); TGW_self, Self-pollinated 1,000-grain weight (g); TGW_OP, Open-pollinated 1,000-grain weight (g);

** *Significant at 1% level*.

Continuous distribution of phenotypic frequency in different environments supports the quantitative inheritance of all observed traits, as expected for quantitative traits. Unimodal distributions of the ICMB 841 × 863B RIL population showed considerable transgressive segregation for FT, PH, PL, TGW (in self and OP seeds) and a significant difference in traits was observed between two extreme RILs, indicating that large variation occurs among the 106 RILs. This magnitude of genetic variation for most of the traits demonstrated that mapping was likely to reveal the underlying QTLs.

### QTL analysis

#### Flowering time (FT)

A total of 22 putative QTLs were detected in E1 and E2 (data not shown), 11 QTLs in each environment for flowering time. A simultaneous fit accounted for 52.9% of adjusted R^2^ in E1 while in E2 adjusted R^2^ was 36.2%. In E1, one QTL on LG7 explained 15.8% adjusted R^2^ with adjusted additive effect of −2.2 d (863B allele conferring earliness) whereas in E2 the QTL on LG3 had the maximum observed adjusted *R*^2^ (21.0%) with the allele from 863B conferring lateness. The LOD in E1 was ranging from 4.3 to 9.8 whereas in E2 it was ranging from 4.2 to 10.6. The *R*^2^-values for individual significant QTLs in E1 and E2 ranged from 17.0 to 34.8% and from 16.8 to 36.9%, respectively. Six QTLs in E1 and five QTLs in E2 were environment specific.

**Figure 1:**
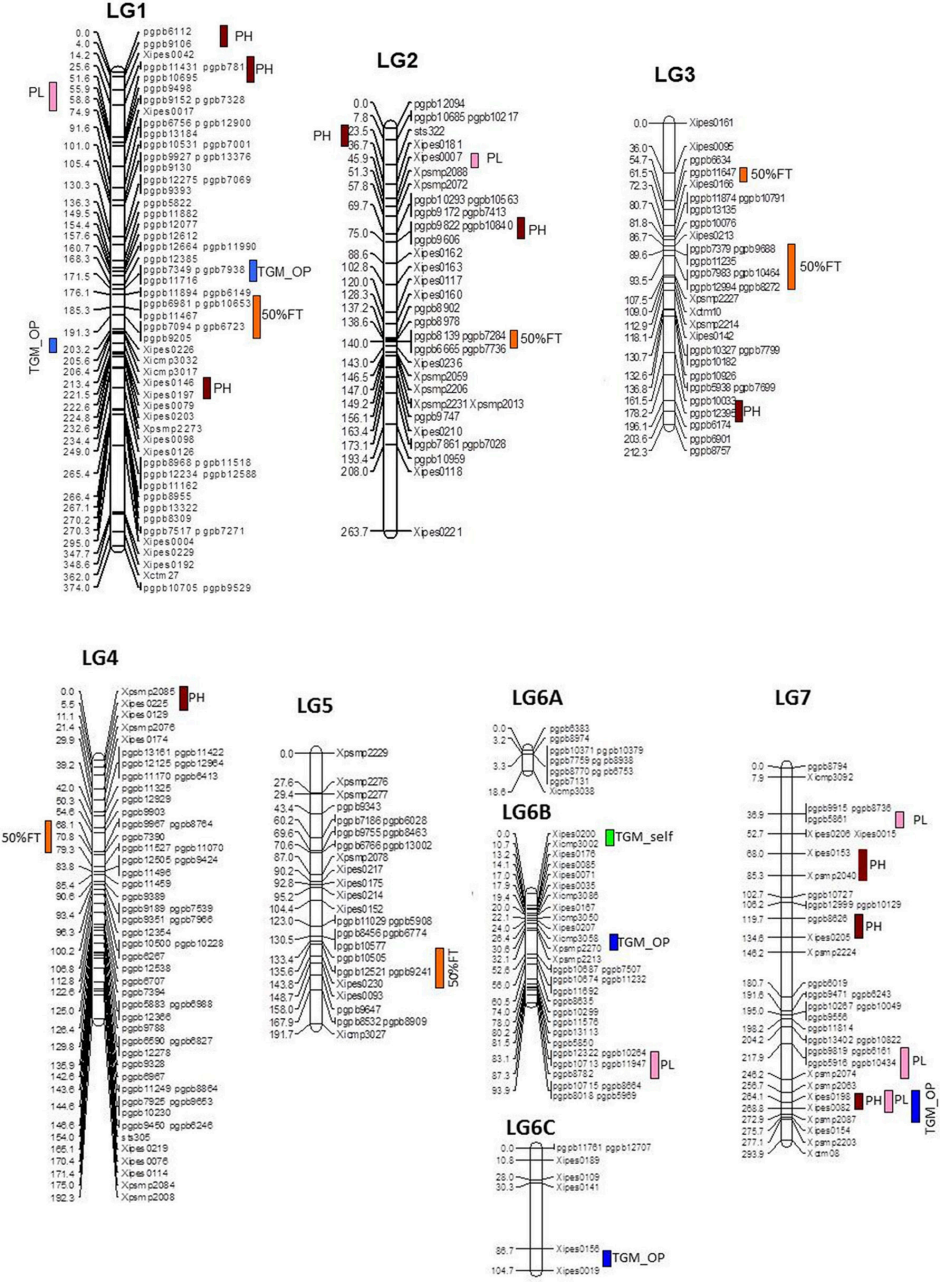
Linkage map with QTL positions for ICMB 841-P3 × 863B-P2 based RIL population (Source of linkage map: Kumar et al., 2016).

During across-environment QTL analysis, PlabQTL (Figure 1, Table 4) detected 6 QTLs, with one each on LG1, LG3, LG4 and LG5 and two QTLs on LG3 were found common in E1, E2 and across environments which altogether explained 23.2% of *R*^2^. One novel QTLs (3/62) was detected in this across-environment QTL analysis. The R^2^ explained by individual QTLs ranged from 23.4 to 48.8%. Similarly, the observed minimum and maximum LOD score were 5.85 and 16.88, respectively. QTLNetwork detected two QTLs in the across-environment analysis with 863B alleles conferring later flowering for both QTLs, with one QTL on LG3 at position 90 cM, the same as identified using PlabQTL while another one on LG6B at position 26 cM was not detected in the PlabQTL analysis.

**Table 4:** Positions and descriptions of QTLs affecting various traits in the (ICMB 841-P3 × 863B-P2)-derived RIL population across the two screening environments at ICRISAT-Patancheru.

**Trait**	**QTL Position^$^**	**Marker Interval**	**Support Interval**	**LOD**	**QEI**	***R*^2^ (%)**	**Additive Effects^#^**
FT	1/166	*pgpb6981-Xipes226*	158–170	8.42	ns	31.9	1.2
	2/136	*Xipes236-Xpsmp2059*	130–140	6.46	ns	25.5	1.8
	3/62	*pgpb11647-Xipes166*	60–68	5.85	ns	23.4	3.1
	3/100	*pgpb7379-Xpsmp2227*	94–106	14.68	^**^	48.8	2.6
	4/56	*Pgpb9967-Xpsmp11527*	54–64	6.05	ns	24.1	0.6
	5/132	*pgpb10505-Xipes230*	126–136	10.05	^**^	36.8	−1.1
PH	1/0	*Pgpb6112-pgpb9106*	0–4	6.4	^**^	30.2	2.9
	1/62	*Pgpb9498-Xipes017*	52–68	8.54	ns	32.3	−3.0
	1/192	*Xipes203-Xpsmp2273*	186–198	8.8	ns	33.0	5.9
	2/4	*pgpb12094-pgpb10685*	2–6	6.67	^**^	28.7	2.1
	2/34	*Xpsmp322-Xipes181*	26–46	6.45	^**^	25.5	2.7
	2/88	*Xipes162-Xipes163*	58–100	5.7	^**^	22.9	3.6
	3/100	*pgpb7379-Xipes2227*	86–106	5.91	^**^	23.6	1.8
	3/206	*pbpb6901-pgpb8757*	202–206	9.83	ns	39.5	−3.1
	4/0	*Xpsmp2085-Xipes225*	0–4	8.87	ns	34.1	6.1
	6B/26	*Xipes207-Xicmp3058*	14–32	5.78	ns	23.2	−0.4
	7/72	*Xipes153-Xpsmp2040*	66–80	9.7	ns	35.8	−6.0
	7/112	*pbpb8626-Xipes205*	104–118	6.42	ns	25.4	−2.9
	7/246	*Xipes198-Xipes082*	240–250	11.13	ns	39.8	−6.4
PL	1/62	*pgpb9498-Xipes17*	54–68	6.93	^*^	27.1	−1.4
	1/168	*Xipes226-Xicmp3032*	166–170	7.27	^**^	28.2	0.7
	1/262	*Xipes4-Xipes229*	250–274	6.42	ns	25.4	0.4
	2/48	*Xipes7-Xpsmp2088*	40–50	7.34	^**^	28.4	1.7
	5/162	*pgpb9647-Xicmp3027*	152–162	6.08	ns	24.4	−0.4
	6B/0	*Xipes200-Xicmp3002*	0–6	8.61	^**^	32.5	−0.4
	6B/56	*pgpb10687-pgpb10299*	52–66	5.94	^**^	24.6	0.9
	6B/74	*pgpb12322-pgpb8782*	72–76	5.95	^**^	26.0	1.1
	7/42	*pbpb9915-Xipes206*	32–56	6.23	^**^	24.7	−1.0
	7/222	*pbpb9819-Xpsmp2074*	214–232	6.65	^**^	26.2	0.2
	7/246	*Xipes198-Xipes82*	240–250	7.84	^**^	30.1	−1.0
TGW_self	6B/10	*Xipes200-Xicmp3002*	4–14	7.36	^**^	28.5	0.8
TGW_OP	1/146	*pgpb7349-pgpb11894*	144–148	8.85	^**^	33.2	0.2
	1/168	*Xipes226-Xicmp3032*	160–170	8.78	^**^	33.0	−0.2
	2/22	*sts322-Xipes181*	18–28	11.28	ns	40.2	−0.5
	2/112	*Xipes117-Xipes160*	106–118	7.55	ns	29.1	−0.1
	2/136	*Xipes236-Xpsmp2059*	134–138	15.45	ns	50.6	0.3
	3/124	*Xipes142-pgpb10327*	110–130	10.97	ns	40.6	0.2
	6B/28	*Xicmp3058-Xpsmp2270*	24–30	21.26	ns	62.1	0.6
	6C/8	*pgpb12707-Xipes189*	0–16	7.32	ns	30.7	−0.2
	6C/88	*Xipes156-Xipes19*	70–98	12.31	ns	43.3	0.3
	7/4	*pgpb8794-Xicmp3092*	0–8	10.24	ns	41.1	0.1
	7/64	*Xipes153-Xpsmp2040*	50–70	8.54	ns	32.2	0.2
	7/198	*pgpb11814-pgpb9819*	186–224	7.24	ns	28.1	0.1
	7/252	*Xipes82-Xpsmp2087*	250–258	10.74	ns	38.7	0.6

$ *Leading number: Linkage group; Trailing number: QTL position in cM*.

# For 1,000-grain weight and panicle length, positive additive effects indicate that the favorable alleles originated from “863B.” For flowering time and plant height, positive additive effects indicate the opposite, i.e., the favorable alleles (early flowering, dwarf plant type) originated from “ICMB 841.”

* Significant at 5% level;

** *Significant at 1% level; ns, non-significant*.

*FT, Time to 50% flowering (d); PH, Plant height (cm); PL, Panicle length (cm); TGW_self, Self-pollinated 1,000-grain weight (g); TGW_OP, Open-pollinated 1,000-grain weight (g*).

QTL-ANOVA exhibited significant QTL × environment interaction (QEI) (Table 6). Out of 12 QTLs detected in the across-environment analysis, 2 QTLs showed significant interaction with the environment (Table 5). This was reflected by deviating QTL effects in the two environments.

**Table 5:** Details of the QTLs detected using QTL Network and data from the RIL population derived from the cross (ICMB 841-P3 ×863B-P2).

**Trait**	**LG/Position^$^**	**Flanking Markers**	**Support Interval**	**Additive Effects^#^**	***R*^2^ (%)**	AE1^*^	AE2^*^	***R*^2^ % (AE)**
FT	3/90	*pgpb7983-Xpsmp2227*	90−104	−1.6	0.154	−	−	−
	7/26	*Xipes0207-Xicmp3058*	24−29	−1.2	0.053	−0.73	0.73	0.041
PH	1/188	*Xipes0203-Xpsmp2273*	186−192	−4.0	0.099	−	−	−
	7/245	*Xipes0198-Xipes0082*	240−253	3.8	0.097	−	−	−
PL	7/247	*Xipes0198-Xipes0082*	244−255	1.1	0.109	−	−	−
TGW_self	6B/58	*pgpb8635-pgpb10299*	51−64	−0.5	0.134	−	−	−

$ Leading number: Linkage group; Trailing number: QTL position in cM

# *Additive effects: a negative value indicates that the allele from 863B increases the trait mean, a positive value indicates that the allele from 863B reduces the trait mean*.

* *AE1, Additive effects in test environment 1 (late Kharif 2009) when Q x E effects are significant; AE2, Additive effects in test environment 2 (summer 2010) when Q x E effects are significant; = No Q x E interaction*.

#### Plant height (PH)

Six and ten putative QTLs influencing PH were identified in E1 and E2, respectively (data not shown). Four QTLs were common in both environments while the remainder were environment-specific. The QTLs in E2 were detected with a minimum LOD score of 6.3 while in E1 it was 4.5. The adjusted *R*^2^-values for E1 and E2 were 56.5 and 62.2%, respectively. The favorable alleles (contributing increased PH) of QTLs on LG7 were contributed by ICMB 841. Two QTLs on LG1, one on LG3 and one on LG7 were common in both environments. The R^2^ values for individual QTLs detected in E1 ranged from 17.9 to 31.0%, while in E2 these ranged from 23.9 to 48.0%. In both environments, the maximum additive effect was the LG1 QTL at position 190. The putative QTL with the largest R^2^ detected in E1 and E2 was on LG1 at position 190 cM.

Of the 13 putative QTLs detected for PH in the across-environment analysis (Figure 1, Table 4), only one was common across analyses of all three datasets. Similarly, 3 QTLs were common between E1 and the across-environment analysis, while 9 new QTLs were detected in the across-environment analysis. The *R*^2^-values of individual QTLs ranged from 22.9 to 39.8%. The LOD values for the QTLs varied between 5.7 and 11.13. Of two QTLs identified using QTLNetwork (Table 5), one on LG1 at position 188 cM was common between the PLabQTL and QTLNetwork analyses and the favorable allele (increasing plant height) was contributed by 863B while ICMB 841B contributed the favorable allele for second QTL. Five QTLs out of 13 showed significant QEI from the PlabQTL across-environments analysis (Table 4), but QTL-ANOVA detected no significant QEI for this trait (Table 5).

#### Panicle length (PL)

In E1, 7 putative QTLs with combined adjusted *R*^2^ = 36.5% were identified for PL, with *R*^2^ ranging from 16.7 to 24.4% (data not shown). Among these QTL, two QTLs detected on LG1 and LG7 showed favorable alleles (increasing PL) inherited from 863B. Similarly, 7 putative QTLs for PL was detected in E2, with *R*^2^-values ranging from 16.8 to 21.9% (data not shown). Only one QTL on LG2 at position 44–48 cM was common in all three analyzed datasets (Figure 1, Table 4).

Seven new QTLs were detected in the across-environment analysis while the remaining 4 QTLs detected were also found in one of the two individual screening environments. The *R*^2^-values ranged from 24.4% to 32.5%, while the combined *R*^2^ was 59.4%. A QTL detected on LG7 at position 247 cM using PlabQTL was also identified by QTLNetwork, with the favorable allele inherited from ICMB 841. QTL-ANOVA for PL showed significant QEI and all except two detected QTLs exhibited significant interaction with the environment (Table 5).

#### 1,000-grain weight (TGW)

In E1 two putative QTLs on LG6B and LG7 were detected for TGW_self, which together accounted for 16.5% adjusted *R*^2^. The major QTL on LG 6B had an *R*^2^ of 31.4% and 8.26 LOD score. The same QTL on LG6B was identified in E2 with a LOD value of 6.32 and *R*^2^ of 25.0% (data not shown). The additive-effects (0.55 g) for the LG6B QTL were same in both screening environments.

A single QTL on LG6B with non-significant QEI was detected forTGW in the across-environment analysis (Figure 1, Table 4). This QTL had R^2^ value of 28.5% and an additive effect of 0.8, but was located in a more distal position than that identified in the individual-environment analyses.

Two QTLs, one each on LG6B and LG7 with LOD values of 6.55 and 4.37, respectively, were found to control the TGW of OP seeds in E1. Together these QTLs provided a total *R*^2^ of 33.5%. In contrast to E1, 11 putative QTLs for open-pollinated 1,000-grain weight were mapped in E2. Together these provided *R*^2^ of 55.8%, with R^2^ values ranging from 16.8 to 42.6%. However, no common QTL for this trait was detected in these two environments (data not shown).

Results of the across-environment analyses for TGW_OP seeds were confusing (Tables 4, 5), and those from PlabQTL appear to be artifacts, as 13 putative QTLs were detected for TGW_OP in the across-environment analysis, of which 6 were new and the remaining 7 were also detected with the E2 dataset, while neither of the putative QTL from the E1 data set was identified in this across-environment analysis. The R^2^ values for individual QTLs ranging from 28.1 to 62.1%. Two of the 13 putative QTLs from this across-environment analysis, showed significant QEI in the QTL-ANOVA (Table 4). However, across-environment analysis for this trait using QTLNetwork failed to detect significant QTLs (Table 5).

### Epistasis

**Figure 2:**
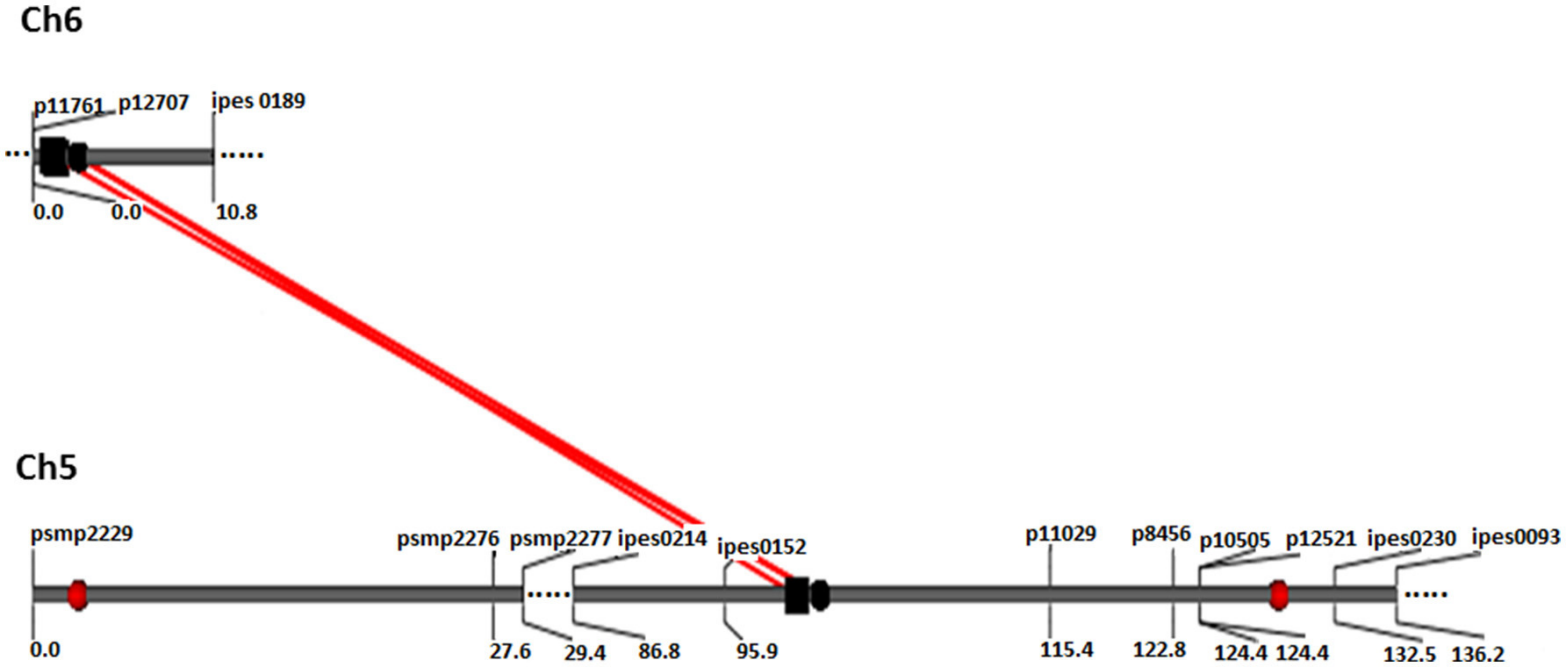
QQ interaction for FT detected using QTLNetwork in and across-environment data from the (ICMB 841-P3 863B-P2)- based RIL population. Red circle represents QTL with an additive effect. Black square and circle represent epistatic QTLs without individual effect, while interacting loci are shown by red colored bar.

A total of 21 digenic interactions were substantiated in the joint analysis (Table 6) while it was 9 (Table 7) and 15 (Table 8) in the E1 and E2, respectively. For example, the panicle length QTLs at 7/222 (LG7 position 222 cM) and 7/42 highly interacted with other QTLs for this trait in the across-environment data analysis (Table 6). The portions of observed phenotypic variation explained by various digenic interactions detected in the across-environment analysis ranged from 18 to 40% for FT and from 13 to 19% for PL. However, QTLNetwork analysis using pooled data identified only 2 significant digenic interactions (Figure 2, Table 9), which was different than interactions detected using PlabQTL (Table 6). All of these putative epistatic effects involved QTLs with no significant main effects. The putative epistatic effects identified by QTLNetwork were not associated with QQE interactions (Table 7).

**Table 6:** Epistatic interactions (additive × additive) for QTLs pairs detected across the two environments with the (ICMB 841-P3 × 863B-P2)-derived RIL population.

**Trait**	**QTL^$^**	**AA effect^$^**	***R*^2^ (%)**	**Epistatic effect^@^**
FT	2/136	4/56	17.90	–
FT	2/136	5/132	22.40	+
FT	2/136	6B/20	22.30	–
FT	4/56	3/100	24.10	–
FT	4/56	4/56	21.00	–
FT	4/56	5/132	37.40	+
FT	3/100	7/178	39.50	+
FT	3/100	7/232	18.90	–
FT	6B/20	6B/72	20.50	+
FT	7/178	7/232	21.30	+
PL	1/62	5/162	12.60	+
PL	1/62	7/222	15.00	–
PL	1/168	5/162	12.70	+
PL	2/48	6B/0	19.30	–
PL	2/48	7/222	16.50	+
PL	5/162	6B/0	15.70	–
PL	6B/56	7/222	11.70	+
PL	6B/56	7/246	13.70	–
PL	6B/74	7/222	15.80	–
PL	7/42	7/222	17.40	–
PL	7/222	7/246	15.40	–

$ *Leading number: Linkage group; Trailing number: QTL position in cM*.

@ *Epistatic effect: a “+” sign indicates that the parental two-locus genotypes have a positive effect on the phenotype, while the recombinants have a negative effect on the phenotype, and a “–” sign means that the parental two-locus genotypes have a negative effect on the phenotype, and that the recombinants have a positive effect on the phenotype*.

*FT, Time to 50% flowering (d); PL, Panicle length (cm*).

**Table 7:** Epistatic interactions (additive × additive) for QTL pairs detected in cross (ICMB 841-P3 × 863B-P2) in test environment 1 (late *Kharif* 2009).

**Trait**	**QTL 1^$^**	**QTL 2^$^**	***R*^2^ (%)**	**Epistatic effect^@^**
FT	2/132	7/192	12.0	–
FT	2/192	7/192	11.4	+
FT	4/58	6B/18	10.1	+
PH	1/60	1/190	8.1	+
PH	1/190	6B/10	9.1	–
PH	3/202	7/254	4.6	–
PH	7/74	7/254	5.5	–
PL	1/58	2/44	5.9	–
PL	7/68	7/154	5.9	–
TGW_OP	6B/16	7/46	14.8	–

$ *Leading number: Linkage group; Trailing number: QTL position in cM*.

@ *Epistatic effect: a “+” sign indicates that the parental two-locus genotypes have a positive effect on the phenotype, while the recombinants have a negative effect on the phenotype, and a “–” sign means that the parental two-locus genotypes have a negative effect on the phenotype, and that the recombinants have a positive effect on the phenotype*.

*FT, Time to 50% flowering (d); PH, Plant height (cm); PL, Panicle length (cm); TGW_OP, Open-pollinated 1,000-grain weight (g*).

**Table 8:** Epistatic interactions (additive × additive) for QTL pairs detected in cross (ICMB 841-P3 × 863B-P2) in test environment 2 (summer 2010).

**Trait**	**QTL 1^$^**	**QTL 2^$^**	***R*^2^ (%)**	**Epistatic effect^@^**
FT	1/168	5/12	10.60	–
FT	1/324	6C/10	10.80	–
FT	6B/20	6B/32	15.20	+
FT	6B/20	6B/72	12.90	–
PH	3/206	7/256	8.30	–
PH	7/244	7/256	8.80	+
TGW_OP	2/108	3/144	16.80	+
TGW_OP	2/108	6B/26	17.30	+
TGW_OP	2/108	6C/14	12.90	–
TGW_OP	2/108	6C/98	13.00	+
TGW_OP	2/138	3/144	18.90	–
TGW_OP	2/138	6B/26	25.20	+
TGW_OP	3/144	6B/26	14.30	–
TGW_OP	3/144	6C/14	9.90	+
TGW_OP	6B/26	6C/98	13.20	–

$ *Leading number: Linkage group; Trailing number: QTL position in cM*.

@ *Epistatic effect: a “+” sign indicates that the parental two-locus genotypes have a positive effect on the phenotype, while the recombinants have a negative effect on the phenotype, and a “–” sign means that the parental two-locus genotypes have a negative effect on the phenotype, and that the recombinants have a positive effect on the phenotype*.

*FT, Time to 50% flowering (d); PH, Plant height (cm); TGW_OP, Open-pollinated 1,000-grain weight (g*).

**Table 9:** QTLs involved in QQ (aa) and QQE (aae) interactions detected by two-locus analysis using QTLNetwork in RIL population derived from (ICMB 841-P3 × 863B-P2) across testing environments.

**Trait**	**QTL^$^**	**Flanking Markers**	**Support Interval**	**QTL^$^**	**Flanking Markers**	**Support Interval**	***R*^2^ (%)**	**Epistatic effect^@^**	**Aae^#^**
FT	5/100	*Xipes152-pgpb11029*	96–109	6C/2	*pgpb12707-Xipes0189*	0–8	0.011	–	–
PL	8/30	*Xipes0141-Xipes0156*	28–41	7/60	*Xipes0153-Xpsmp2040*	54–66	0.100	+	–

$ *Leading number: Linkage group; Trailing number: QTL position in cM*.

@ *Epistatic effect: a “+” sign indicates that the parental two-locus genotypes have a positive effect on the phenotype, while the recombinants have a negative effect on the phenotype, and a “–” sign means that the parental two-locus genotypes have a negative effect on the phenotype, and that the recombinants have a positive effect on the phenotype*.

# *aae, Additive × Additive × Environment interaction effects: a “–” sign indicates that these interaction effects were not significant*.

*FT, Time to 50% flowering (d); PL, Panicle length (cm*).

## Discussion

Globally, pearl millet is a key food, feed and fodder crop for the semi-arid and arid regions. It is a nutritious climate change ready crop, and is one of the cheapest sources of protein, carbohydrates, and minerals. Pearl millet has many health benefiting properties and is accessible to the poor (Anuradha et al., 2017). However, little information is available in pearl millet for important adaptation and agronomic traits like FT, PH, PL, and grain weight. Therefore, this study was attempted to map QTLs for the above mentioned traits in a RIL population of the ICMB 841-P3 × 863B-P2 cross.

### Mean performance

There was a significant variation for the studied traits in pearl millet. Early maturity, large grain size, and compact panicles are some important attributes of the *Iniadi* landrace (Andrews and Kumar, 1996) observed in lines such as 863B-P2. Analysis showed that parents exhibit substantial differences for FT, PH, and TGW traits. The parent ICMB 841 and RILs were shorter in the late *Kharif* environment, while the parent 863B exhibited similar height in both environments. However, a difference between parents and RILs for PH seems to be under strict genetic control with GEI (Poncet et al., 2004). Marginal differences over the environments could be because of the variations in growth period in different environments. Moreover, the photoperiod-temperature response of flowering is the most likely factor responsible for this, as the shorter day length and moderate temperatures during the late *Kharif* sowing were expected to induce early flowering, resulting in shorter plant height (and shorter panicle length). Rahman et al. (2009) also recorded enhanced plant growth, flowering, and maturation in higher temperature in wheat. High temperature increases root growth allowing plant roots to explore a larger volume of soil for moisture and nutrients, therefore relative growth rate (RGR) and net assimilation rate (NAR) increased significantly in pearl millet (Ashraf and Hafeez, 2004). Plant height of the RILs was skewed toward more height in late *Kharif* while in Summer a big proportion of RILs was beyond the height of the taller parent and showed transgressive segregation. This indicates that higher temperatures during the Summer season increased plant height through increased internode length and PL, and presence of higher frequency of alleles for PH and PL.

PL is an imperative component character to achieve good panicle and grain yield. The mean panicle length was slightly higher in summer season (20.3 cm) than in late *Kharif* (18.5 cm). Similar results were observed by Baskaran (2007) in pearl millet where summer was more favorable season for the trait. The trend is comparable to that for plant height indicating the correlated response of this trait as panicle length is a part of plant height.

For TGW_self, late *Kharif* season was more favorable than summer as the population showed higher TGW in late *Kharif*. Stem reserves is a significant carbon source during grain filling. In late *Kharif* plant height was less than in Summer, which could partially explain the higher 1,000-grain weight in late *Kharif* because more assimilate will deposit in the sink instead of the source. Under Summer conditions higher consumption and remobilization of the stem reserves is an important supporting phenomenon that can mostly compensate grain yield reduction (Palta et al., 1994). But this phenomenon was not apparent for the parents where parent 863B with almost same height in both seasons but showed a significant selfed seed TGW difference between the seasons. In contrast to this, parent ICMB 841 showed the reverse phenomenon where height differences did not affect TGW of selfed seeds. This is a clear example of genotype × environment interaction, which shows that plant height was not strongly related to selfed seed TGW across environments.

The OP seeds have more relevance to increased productivity compared to the selfed seeds. Therefore, we compared the TGW of self and OP seeds in the individual as well as across environments (Table 1). The mean TGW values of selfed and OP seeds (for either of the two parents or for the RIL population as a whole) in the joint analysis did not differ significantly, but comparisons of the OP seeds from the individual environments revealed that Summer is the more favorable season for the 1,000-grain weight of OP seeds. Many researchers have measured xenia, the effect of pollen source, for seed weight in maize (Pletsch-Rivera and Kaeppler, 2007). In the present study, TGW values differed between the two types of seed samples. This may be due to xenia, which might have higher activity in the summer season resulting in higher TGW of OP seeds. Briefly, the phenotypic characterization of the population showed the presence of ample genetic variability between parents. This variability will offer chances of recovering desired recombinants with opportunities to map QTLs for studied traits. The observed variability can be exploited to breed high-yielding pearl millet with early flowering and bold seeds.

### Genetic variance, G × E interaction and heritability

The variation and heritability of a character decide the consistency and reliability of QTL mapping (Kearsey and Farquhar, 1998). The analysis of variance suggested that genotypic variance was significantly higher in both environments, and higher H^2^ supported effective QTL mapping. Low GEI for traits indicated that it might be less challenging to select the superior genotypes. However, many previous researchers have recorded significant GEI for FT, PH and PL (Ali et al., 2001; Baskaran, 2007).

Heritability estimates are always unique to the population under study, the growing conditions, the traits observed (and the methods by which they are observed), and the experimental design used. Heritability is an index of the efficacy of transmission of traits from parents to their offspring (Falconer, 1989). In an across-environment analysis of the (ICMB 841 × 863B)-based RIL population, except TGW_OP, heritability estimates in present study were reasonably good and suggest that the expression of studied traits was not greatly influenced by the environment or G × E interactions (Table 3).

### Estimation of correlation coefficients

Before starting breeding, knowledge of association among traits enables the breeders in deciding a suitable selection/breeding program criterion for simultaneous genetic improvement of complex and associated traits (Govindaraj et al., 2009). High levels of correlation of the phenotypic data indicate the co-localization of QTLs for different traits (Paterson et al., 1991). Since phenotypic correlations include both genotypic and environmental components, the genotypic correlations were also determined for studied traits. Higher genotypic correlations compared to phenotypic correlations suggested less interaction between genetic make-up of traits and environmental conditions. Higher genotypic correlation coefficients than phenotypic coefficients also indicated inherent relationships between the traits studied. This finding is in agreement with Khairwal et al. (1999) and Ezeaku and Mohammed (2006).

The correlation between plant height and flowering time was significant, and in agreement with the previous reports (Anarase et al., 2001; Baskaran, 2007). Further, panicle length and plant height showed significant positive correlations at both genotypic and phenotypic levels. However, it is interesting that while panicle length was significantly positively correlated with plant height, it was negatively correlated with flowering time (although these negative correlations were seldom significant). The relationship between PH and FT is especially important in members of Poaceae where apical growth is terminated with flowering (Lin et al., 1995). Domestication and breeding of cereals belonging to tropical origin has mainly focused on the selection of genotypes with dwarfness and day-neutral flowering. Virtually, the Poaceae evolution occurred in the wild 65 million years ago. During this period, increased height afforded a competitive advantage for light interception and seed dispersal, while short-day flowering harmonized plant development with the availability of water in the semi-arid centers of origin (Harper, 1977). All mapping studies that showed correlations between these two traits have also shown specific QTL regions that influenced both traits (Lin et al., 1995). In the present study, one QTL was common for these two traits, which may explain the cause of positive correlation between FT and PH.

Generally, during grain filling stage, photosynthesis contributes a major portion of the final grain carbon and carbohydrate volume (Murchie et al., 2002). The rest of carbon amount is contributed from remobilization of stored carbohydrate from aerial plant parts (Yoshida, 1981; Watanabe et al., 1997). In the present investigation, genotypic and phenotypic correlations between selfed seed TGW and flowering time was significantly negative in the summer 2010 evaluation (data not shown), and also significantly negative in the across-environment analysis (Table 3). This is perhaps a result of higher evaporative demand later in the hot summer season resulting in forced maturity and incomplete grain filling in later-flowering entries, as was observed by Baskaran (2007), but the negative correlations were weaker for flowering time and OP seed TGW. Yadav et al. (2003) also found a negative correlation between FT and grain yield. Higher yield production ability is a foremost prerequisite for any crop cultivar. Therefore, during breeding to enhance expression of any other traits, special consideration should be paid to circumvent negative impacts on grain yield (Peleg et al., 2009).

A positive significant correlation was detected between selfed seed TGW and plant height at both the genotypic and phenotypic levels in across-environment analyses. However, none of these correlations were strong enough to seriously hinder or help the simultaneous improvement of both traits. The negative association of panicle length with TGW could be attributed to linkage or to yield component compensation (Vengadessan, 2008). The correlations of TGW_OP seeds with plant height and panicle length followed the same trends as detected for TGW of selfed seeds. Plant height and panicle length in addition to grain yield and crop cycle length are traits considered by farmers in choosing pearl millet cultivars. Shorter plants are desirable in particular environments because of their tolerance to strong windy conditions that could cause lodging of taller plants. Positive and significant associations with plant height were observed for panicle length. The correlations between these two traits indicate that taller plants tend to bear longer panicles. Similar significantly positive correlations in pearl millet were reported by Baskaran (2007), Vengadessan (2008) and Govindaraj et al. (2009).

### Mapping quantitative trait loci (QTLs)

#### QTLs for flowering time

Flowering time, a “drought escape mechanism,” is a key trait which is responsible for adaptation of pearl millet to drought conditions. QTLs for flowering time were identified on 4 chromosomes of the cross ICMB 841 × 863B. Except for the QTL on LG3, these QTLs were also identified in previous studies. In the current experiment, a significant QTL for flowering time was detected on LG2- linkage group harboring PHYTOCHROME C gene (*PHYC*) which has a significant association with flowering time (Saïdou et al., 2009). The large additive effects detected in the joint analysis suggested a very broad range of flowering time in this population. Large additive effects may be due to the segregation distortions present in the RIL population. The QTL on LG5 reported by Baskaran (2007) was likely to be the same as the LG5 QTL recorded in the current study. Considering the many earlier reports (Hash et al., 1995; Nepolean, 2002; Yadav et al., 2002), the detected positions of flowering time QTL on LG4 is robust. The QTL on LG4 did not show QEI, while two QTLs with high R^2^ showed high interaction with the environment. Using another population, Hash et al. (1995) also reported flowering QTL on LG1 with small effect.

#### QTLs for plant height

It was interesting to observe that high numbers of putative QTLs were found for PH distributed across the whole genome, a trait considered to be a relatively simply inherited due to the involvement of few loci. Poncet et al. (2000, 2002) mapped QTLs for plant height on all pearl millet linkage groups except LG3 and LG4. Similarly, Nepolean (2002) located a major gene for plant height, *d2*, on LG4, and Baskaran (2007) detected one QTL on LG3. Vengadessan (2008) suggested that QTL on LG3 may be considered as another dwarfing locus. QTL for PH was also mapped on LG1 by Azhaguvel et al. (2003) is similar to the present study. Vengadessan (2008) mapped six plant height QTLs on LG1, LG2, LG3, LG4, and LG5. In the present study, one QTL on LG3 was consistent without any QEI. Another QTL on LG7 also appeared consistent, but it showed a small position change. In the present study, perhaps due to the small size of the (ICMB 841 × 863B)-based RIL population, no consistent plant height QTL on LG4 could be detected. Significant QEI for plant height (and/or flowering time) could be the reasons for differences in plant height QTLs detected in late *Kharif* and Summer seasons. From previous reports, it can be concluded that expression of traits like plant height with high heritability are also controlled/regulated by a large number of genes/loci. This suggests that more careful study must be carried out during dissection of the inheritance pattern of such traits. One QTL on LG3 for (ICMB 841 × 863B)-based RILs was common between flowering time and plant height, and could partially explain the positive correlations between these traits; however, a larger portion of common QTLs was expected. The detected QTL can be helpful to alter the value of pearl millet forage. According to Burton and Forston (1966), five dwarfing genes are documented in pearl millet, with the wide use of only d2 locus in breeding. Though, this locus causes a yield penalty due to pleiotropic association with low grain weight (Bidinger et al., 2001), it can be overwhelmed by employing favorable genetic background (Vengadessan, 2008) and exploiting other dwarfing loci as detected in the present study.

#### QTLs for panicle length

Panicle length is an important yield contributing trait. Three major QTLs for panicle length, one each on LG1, LG2, and LG6B, were identified in the joint analysis of the two-season (ICMB 841 × 863B)-based RILs data sets. Earlier studies demonstrated that QTLs influencing the expression of panicle length are present on LG1, LG2, LG4, LG6, and LG7 (Poncet et al., 2000, 2002; Nepolean, 2002; Baskaran, 2007; Vengadessan, 2008). Two QTLs, one each on LG1 and LG7, were common for plant height and panicle length, which partially explained the positive correlations between these traits.

#### QTLs for grain weight

Grain weight is an important component for grain yield. A single major effect QTL for TGW on LG6B of the (ICMB 841 × 863B)-based RILs was identified in the joint data analysis of the present study. This has also been reported by Baskaran (2007). In E1, one additional putative QTL on LG7, as detected by Baskaran (2007), was also detected with a minor effect. Likewise, one putative major QTL for the TGW of OP seeds was detected on LG6B along with large numbers of season-specific putative QTLs. Similarly, Bidinger et al. (2007) detected grain weight QTLs on LG1, LG2, LG3, and LG6. Though, Yadav et al. (2002) reported QTLs for 100-grain weight on LG2 and LG7. The QTL of this study for TGW did not correspond to the locations informed earlier. However, after the availability of pearl millet genome sequence, potential candidate genes can be identified.

Overall, the incongruities in the QTL positions detected in the present study with the earlier reports may be due to differences like population size, population type, parental genomic background, type of markers, etc.

There may be an overestimation of *R*^2^ and LOD value of QTL reported in current research. However, these QTL can be validated in different populations, association mapping and large sized population from same parents. ICRISAT has developed a large-sized bi-parental population of ICMB 841 × 863B cross to further validate the results reported in this as well as earlier experiments on this population.

#### Epistasis

In the present study data are based on recombinant inbred lines and hence only additive × additive epistatic interactions could be measured. In environment-wise QTL identification for the (ICMB 841 × 863B)-based RILs using PlabQTL, except for TGW_self, epistasis was detected for all traits studied. During joint analysis across the two environments for this population, FT data showed equal numbers of signs (+ and −) indicating that recombinant two-locus genotypes and parental two-locus genotypes were equally likely to have a negative effect on trait expression that would decrease flowering time. In contrast, for panicle length negative signs predominated, indicating that recombinant two-locus genotypes tended to have a positive effect on panicle length. Joint analysis across both environments data sets revealed apparent epistasis for flowering time and panicle length. However, a caution should be exercised while considering results of epistatic interactions among QTLs due to the inadequate size of the mapping population (Gallais and Rives, 1993).

QTL × QTL interaction (QQI) and QTL × QTL × environment interaction (QQEI) analysis with QTLNetwork showed none of the major QTLs was involved in QQI/QQEI. The effects of epistatic QTLs on phenotypic variation were insignificant and these interactions can be ignored in genomics-assisted breeding. The probable cause for null or little epistasis may be due to the intra-specific population using well-adapted parental lines (Kumar et al., 2016) used in this study. The intra-specific cross reduced the chances of interruption of co-adapted epistatic genomic blocks (Melchinger et al., 1998).

## Conclusions

Breeding early, bold seeded lines with higher grain yield is one of the most important breeding objectives for pearl millet globally. The measurable genetic variation for important agronomic and adaptation traits such as flowering time, plant height, panicle length and grain weight as demonstrated in the present research was exploited to detect major effect stable QTLs across two environments. Since the reported QTLs are from a relatively smaller RIL mapping population with lower marker density, the QTL effects might have been under-or overestimated. Therefore, validation of these QTLs should be performed in diverse genetic backgrounds, before they can be considered as reliable targets for marker-assisted selection (MAS) in pearl millet hybrid and varietal breeding programs for enhancing adaptation and grain yield.

It will also be interesting to further dissect the co-localized flowering time and plant height QTLs reported in this study. It may throw light on the role of possibly common genes controlling flowering time, plant height, and in evolution and adaptation of pearl millet.

## Author contributions

CH and RS designed research. SK, RS, CH, TN, and GS performed research. RS supervised data analysis and interpretation. SK analyzed the data. RS, SK, CH, CS, RY, and MM wrote the paper. RS critically revised the paper for final publication.

### Conflict of interest statement

The authors declare that the research was conducted in the absence of any commercial or financial relationships that could be construed as a potential conflict of interest.
